# Avalanche transceiver search times during avalanche companion rescue – A prospective randomized single-blinded cross-over simulation study

**DOI:** 10.1016/j.resplu.2025.101065

**Published:** 2025-08-19

**Authors:** Bernd Wallner, Simon Woyke, Manuel Winkler, Fabio Caramazza, Ivo B. Regli, Gabriel Putzer, Giacomo Strapazzon, Markus Falk, Hermann Brugger, Katharina Hüfner, Peter Mair

**Affiliations:** a Department of Anaesthesiology and Intensive Care Medicine, Innsbruck Medical University Hospital, Medical University of Innsbruck, Anichstrasse 35, 6020 Innsbruck, Austria; b Institute of Mountain Emergency Medicine, Eurac Research, Via Ipazia 2, 39100 Bolzano, Italy; c Department of Cardiac Anaesthesiology and Intensive Care Medicine, Deutsches Herzzentrum der Charité (DHZC), Berlin, Germany; d eScience, Sonnenstrasse 11, I, 39031 Bruneck, Italy; e Department of Psychiatry, Psychotherapy, Psychosomatics, and Medical Psychology, University Clinic for Psychiatry II, Innsbruck Medical University, Anichstrasse 35, 6020 Innsbruck, Austria; f Department of Medicine – DIMED, University of Padova, Padova, Italy

**Keywords:** Avalanche rescue, Avalanche transceiver, First responder, Voice navigation, Training

## Abstract

**Background:**

The aim of this study was to analyse the influence of voice navigation in a new generation of transceivers on the time and success rate of transceiver search in a simulated avalanche rescue scenario.

**Methods:**

Fifty participants performed two randomized test runs, using two different transmitters with and without voice navigation. Primary outcome was success rate, total transceiver search and total location time, secondary outcome parameters comprised predefined time intervals (coarse search time, fine search time, probing time) and deviations from the recommended standard search procedure.

**Results:**

The study shows comparable results in success rate, total transceiver search time (111.1 s ± SD84.3 s) and total location time (134.4 s ± SD 112.6 s) for trials with and without voice navigation. Wrong initial search direction was corrected earlier in trials with voice (49.6 ± 6.9 s versus 93.7 ± 18.4 s p = 0.011). Participants were significantly faster in the second search trial (first 203.0 s versus 143.2 s; p = 0.002), a learning effect, more pronounced with voice navigation (voice 119.8 s vs. no-voice 164.8 s; p = 0.014). Deviations from the recommended standard search procedure were common (55 % of trials), without any difference between voice and no-voice navigation.

**Conclusions:**

This study demonstrated a wide inter-individual variation in total transceiver search times and total location time in participants without experience. Voice navigation did not result in a significant reduction of transceiver search times but led to a faster correction of a wrong initial search direction and improved the learning effect significantly in second trials. Voice navigation could possibly optimize performance in participants without prior experience in stressful situations.

## Highlights

• Participants demonstrated a wide inter-individual variation in avalanche transceiver search.
• Voice navigation significantly improved the learning effect.
• Voice navigation led to a faster correction of a wrong initial search direction.
• Voice navigation could possibly optimize performance in stressful situations.
• Avalanche rescue training should also concentrate on the avoidance errors during fine search.

## Introduction

Avalanche accidents pose a significant risk of fatality for off-piste and backcountry skiers and snowboarders in mountainous regions. The leading cause of mortality in avalanche victims is asphyxia after critical burial.^1^, ^2^, ^3^, ^4^, ^5^ Critical avalanche burial defines the circumstance when an avalanche victim’s head and chest are completely covered by avalanche debris, in this case the chance of survival is about 50 %.^6^ Consequently, rapid extrication to avoid cardiac arrest from asphyxia is a major determinant of survival in avalanche accidents. The chance of survival is ∼ 90 % for critically buried victims extricated within 10 min, but drops rapidly to about 30 % when burial time reaches 30 min.^7^, ^8^ If no part of the victim is visible on the surface of the avalanche, companion rescue with avalanche transceivers is the only possibility to locate and extricate the victim within minutes after burial. Organised rescue will normally arrive at scene only after this first critical time period.^7^, ^9^, ^10^, ^11^ During the decade from 1990 to 2000, major improvements in transceiver technology and user usage and training improved the success rate of companion rescue and reduced the time necessary to locate a completely buried avalanche victim.^8^, ^12^, ^13^, ^14^

One recent technological improvement was the introduction of voice navigation. This new technology guides the user through the search process with clear verbal instructions and corrects the search process in case of deviations from the recommended approach. Voice navigation should thereby assist the user and improve avalanche transceiver search. However, the actual influence of voice navigation on the rate of successful location and search time has not been evaluated so far.

The primary aim of this prospective randomized single-blinded cross-over study was to analyse the influence of voice navigation on time and rate of successful victim localization of transceiver search in a simulated avalanche rescue scenario.

## Material and methods

This prospective mixed methods crossover study was part of a larger study on the effect of verbal commands on avalanche rescue.^15^ The ethics committee of the Medical University of Innsbruck, Austria, was contacted and considered a rigorous ethical evaluation unnecessary (inquiry to the Ethics Committee on 23.12.2021). After being informed in detail about the study aims and procedures, all participants provided informed consent prior to study participation. The study was conducted on two days in March 2022.

### Participants

Participants were 50 student volunteers from the Medical University Innsbruck, age > 18 years (50 % female and 50 % male, mean age 24.0 ± 3.6 years) regularly performing winter sports, but with no formal training or practical experience in avalanche rescue and transceiver search. All 50 participants gave written informed consent. Students with training or experience in avalanche transceiver search were excluded for homogeneous sampling.

### Study site and search simulation

**Fig. 1:**
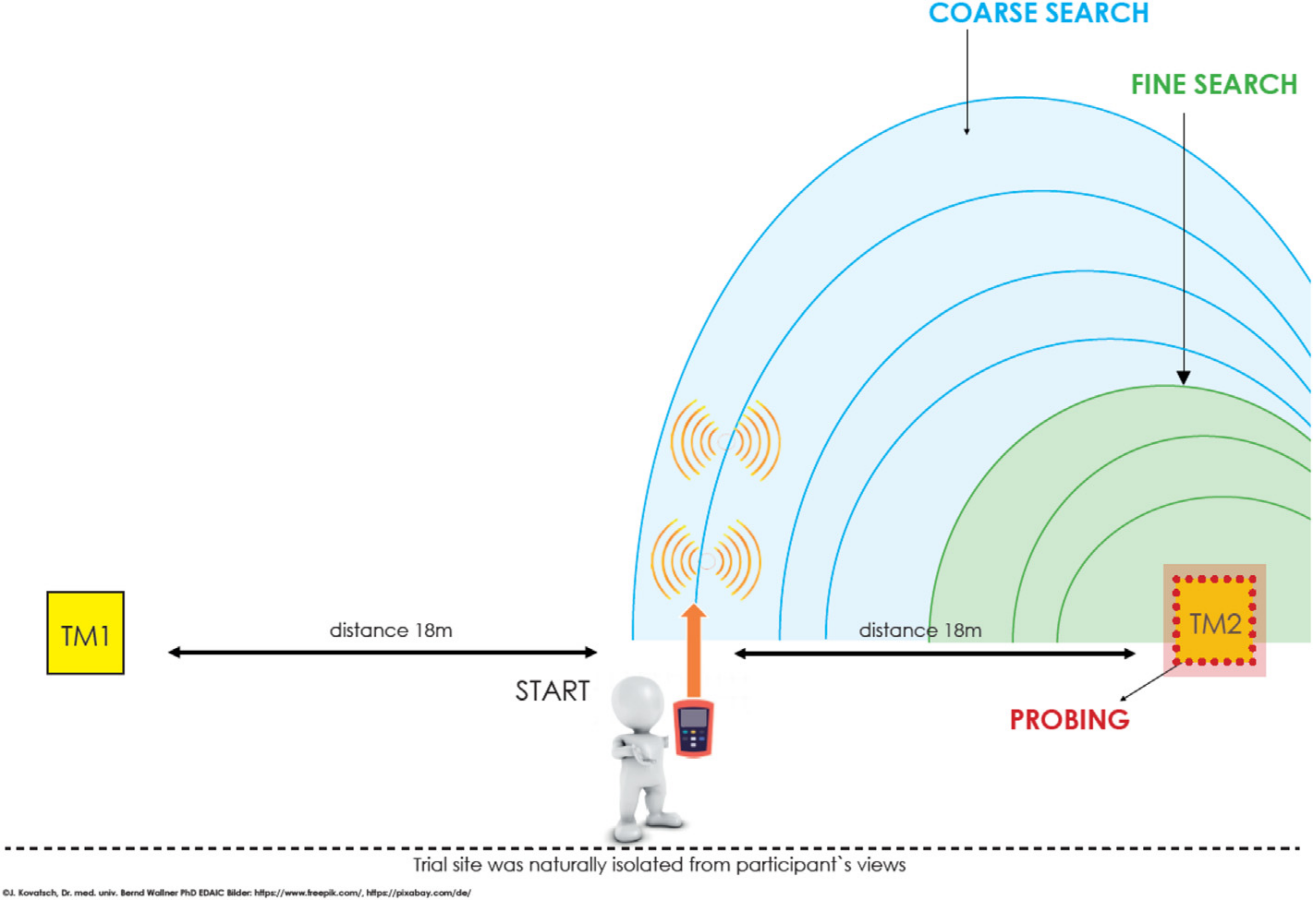
Graphical display of the trial site setup. Either transmitter TM1 or TM2 were randomly activated. Participants started after verbal induction of stress in each trial at the same spot in between the two transmitters. Each trial began in coarse search (blue area) before participants reached fine search (green area) and then commenced probing. (For interpretation of the references to colour in this figure legend, the reader is referred to the web version of this article.)

The study site was located at 2450 m a.s.l. in the Kühtai skiing area, Tyrol, Austria. An artificial avalanche field was prepared on a level surface with two transmitters buried in a depth of 100 cm under the snow-surface (RTX457 Mobile Avalanche Transceiver Training System; developed by Girsberger Mountain Rescue Technology). Both transmitters were placed at a distance of 18 m from the starting point, which was located exactly between both transmitters (see Fig. 1). Transmitters were attached to wooden panels coated with elastic material on the upper site to mimic an avalanche victim’s trunk for probing. The avalanche transceiver “Diract Voice” (Ortovox, diract voice avalanche transceiver, Ortovox Sportartikel GmbH, Rotwandweg 3a, D-82024 Taufkirchen, Germany^16^ was used, according to randomization either with or without activated voice navigation. A conventional avalanche probe (Ortovox Sportartikel GmbH, Rotwandweg 3a, D-82024 Taufkirchen, Germany) was used for probing.

### Study protocol

One week prior to the trials, a link to a ten-minute video with structured instructions on how to perform an avalanche search and how to adequately use the avalanche transceiver and probe was sent out to each participant. It was recommended to watch the video at least once, but participants were allowed to watch the video repeatedly.

The video was manufactured by the Austrian Alpine Club (Österreichischer Alpenverein).^17^ During the investigation the trial site was visually isolated so participants could not inspect the test setup until personal involvement. The participants were blinded to the entire test setup, the expected task, the location of the transmitter, the search direction, and the assigned transceiver technology (with and without voice navigation). To prevent communication between the participants, they were separated from each other before and after the tests. Immediately before the trial, participants were exposed to a hypothetical scenario, such as them being part of a skiing team and one member of the team being completely buried in the avalanche. The scenario was presented to the participant in a way to evoke stress as during a real-life avalanche rescue situation.

**Fig. 2:**
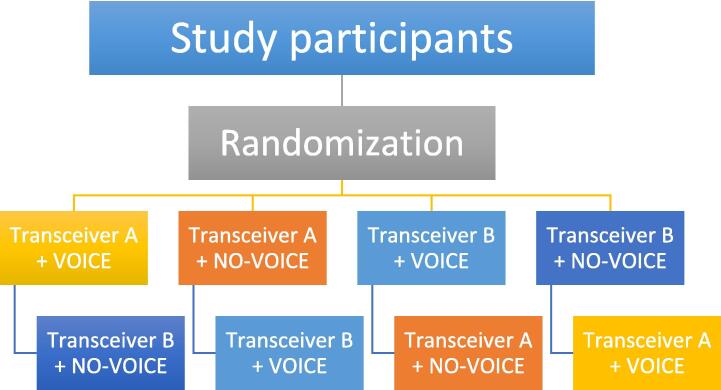
The randomization was performed in the depicted manner. Participants either started searching for transceiver A transceiver or B and with or without voice navigation, and were then switched to the other transceiver and the other navigation, respectively.

All participants performed two test runs, including one search with and one without voice navigation and one search towards the left and one towards the right transmitter. Sequence of activation or non-activation of voice navigation and choice of transmitters (left or right) was fully balanced and randomized (see Fig. 2). Trials were stopped when the participant hit the transmitter panel with the probe and called out “successful hit”. Trials were also ended after a total trial time of 10 min or after three incorrect hits with the probe. One of the investigators accompanied the participant and documented typical errors and deviations from the recommended search strategy using a predefined list (see below).

The primary outcome of this prospective randomized single-blinded cross-over study was to analyse the influence of voice navigation on time and rate of successful victim localization of transceiver search in a simulated avalanche rescue scenario. Secondary outcome parameters were predefined time points during the course of the avalanche search (coarse search time, fine search time, probing time), as well as the incidence and significance nine commonly observed errors or deviations from the recommended standard search approach.

### Measurements

For each search trial the following time intervals were obtained: i) coarse search time, defined as the interval from the start of the trial to the beginning of the fine search, ii) fine search time, defined as the time interval from the start of the fine search until probing, and iii) probing time defined as the interval from the start of probing until successfully hitting the wooden transmitter panel. Total transceiver search time was defined as the sum of coarse search and fine search time. Total transceiver search time combined with probing time added up to total location time. To further differentiate between fast and slow trials, we defined a cut-off value of 180 s of total location time. This cut off value corresponds to three times the maximum search time observed in this search scenario for trained persons.

Coarse search time is the first time interval when the participant is moving quickly towards the transmitter guided by a moving arrow on the transceiver’s display showing the correct direction. It comprises the time interval between first signal detection and the moment when the moving arrow disappears on the transceiver and is replaced by a cross-marking. Fine search time is the time when the participant is cruising slowly with the transceiver close to the snow surface in right angular movements until the transceiver shows minimal distance from the transmitter. Fine search time comprises the time interval between appearance of the cross-marking on the transceiver display and the start of probing. The minimal distance displayed by the transceiver when the participant started with probing was also recorded, as well as the number of probe hits necessary for successful localization of the transmitter panel.

Using a predefined list, one investigator checked for the presence of nine commonly observed errors or deviations from the recommended standard search approach. The only deviation looked for during coarse search, which was “wrong direction of coarse search” and described the problem of choosing the wrong direction away from the transceiver after initial signal detection. Deviations characterizing “inadequate fine search” were “jumpy transceiver handling“; “moves too fast in fine search”; “no crossing in fine search”; and “fine search not near to the snow surface”. Deviations indicating “inadequate probing” included “probing radius too small”; “uncoordinated probing”; “probe radius too large”; and “insufficient probing depth”.

### Statistical analysis

Data from a pilot study revealed an average search time of 120 s with a standard deviation of approximately 60 s. To detect a practically relevant difference of 20 s between methods, with two repetitions per subject, a sample size of 50 subjects would be necessary to achieve an 80 % power, with an alpha level of 5 %. Group mean differences were evaluated using the *t*-test, while pre- versus post-comparisons employed the paired *t*-test. Group comparisons in counted data were conducted using the chi-square test, while time to rescue was analysed using the Kaplan-Meier estimator, with group comparisons performed via the log-rank test. Continuous data was presented as mean and standard deviation or as median with range, as appropriate, while frequencies were utilized for counted data. Statistical analyses were carried out using SPSS 29, with significance set at a two-sided p-value less than 0.05.

## Results

Ninety-five of all 100 search trials were completed, two were terminated because the maximum trial time of 10 min was exceeded (one using voice, one no-voice), and three because of probing failure after three erroneous hits (one using voice, two no– voice).

### Comparison of search times in voice and no-voice trials

Means for total transceiver search time, coarse search time, fine search time, probing time and total location time for trials with and without voice navigation are shown in Table 1. The study did not find a significant difference in any of the time intervals obtained for trials with and without voice navigation.

**Table 1:** Means ± SD in seconds for coarse search time, fine search time, probing time, total transceiver search time and total location time are shown for the first (Trial 1) and second trial (Trial 2) and in comparison for trials with and without voice navigation. There was no significant difference in any of the time intervals obtained for trials with and without voice navigation.

	All trials		Trial 1		Trial 2	
Times	**VOICE**	**NO VOICE**	**VOICE**	**NO VOICE**	**VOICE**	**NO VOICE**
Coarse-search time	55.6 ± 7.4	66.8 ± 11.1	62.2 ± 48.4	70.3 ± 68.5	48.3 ± 52.0	63.6 ± 70.3
Fine-search time	48.3 ± 6.0	51.4 ± 9.2	56.4 ± 47.3	53.3 ± 35.3	39.6 ± 26.7	49.7 ± 58.9
Probing time	54.0 ± 13.1	59.0 ± 13.2	74.4 ± 102.7	71.1 ± 85.7	31.8 ± 44.9	47.9 ± 68.6
Total transceiver time	103.9 ± 67.3	118.2 ± 97.1	118.6 ± 67.8	123.6 ± 89.2	87.9 ± 65.9	113.3 ± 107.2
Total location time	157.9 ± 15.6	177.2 ± 18.2	193.0 ± 25.6	194.7 ± 26.7	119.7 ± 14.7	161.2 ± 25.6

### Total transceiver search times and total location time

Total transceiver search time for all search trials was mean 111.1 s ± SD 84.3 s, and total location time was 134.4 s ± SD 112.6 s. There was a great individual variation in total transceiver search time as well as total location time among the study participants during both, voice and no-voice search trials. A total of 68 % of all trials were completed within 180 s (fast trials) (mean 106.1 s ± SD 35.3 s). 32 % participants completed the trial after >180 s (slow trials) (mean 298.6 s (± SD 108.3 s)). Slow search trials were equally frequent with and without voice navigation (p = 0.254), but less frequent in participants performing their second trial (84.0 % in first versus 16.0 % in second search trial (p = 0.019).

### Minimal distance and probing

The minimal distance revealed by the transceiver after fine search was not different between groups using voice navigation (mean 1.08 m ± SD 0.26 m) versus those without voice navigation (mean 1.07 m ± SD 0.26 m). In 60 % of trials the target was located on the first, in 28 % on the second, and in 9 % on the third hit. The use of voice navigation also had no impact on probing time or success (Table 1, Table 2).

**Table 2:** Incidence of deviations from standard search technique. No significant difference in the incidence of error in the voice group compared to no-voice. The problem “wrong direction of coarse search” had a significant impact on the coarse search time (*) (p < 0.001) and was corrected earlier using voice navigation (49.6 ± 6.9 versus 93.7 ± 18.4p = 0.011).

	Type of error	ALL	VOICE	NO VOICE
		**n = 100**	**n = 50**	**n = 50**
Coarse-search time	wrong direction of coarse search	n = 55 (55 %)	n = 28 (56 %)*	n = 27 (54 %)

Fine-search time	Too fast at 3 m	n = 24 (24 %)	n = 12 (24 %)	n = 12 (24 %)
	Sudden moves in fine search	n = 24 (24 %)	n = 9 (18 %)	n = 15 (30 %)
	No crossing in fine search	n = 35 (35 %)	n = 18 (36 %)	n = 17 (34 %)
	Fine search not on the snow surface	n = 13 (13 %)	n = 5 (10 %)	n = 8 (16 %)

Probing time	Probing radius too small	n = 33 (33 %)	n = 14 (28 %)	n = 19 (38 %)
	Probing without any scheme	n = 30 (30 %)	n = 13 (26 %)	n = 17 (34 %)
	Probe radius too large	n = 15 (15 %)	n = 7 (14 %)	n = 8 (16 %)
	Probing not deep enough	n = 19 (19 %)	n = 7 (14 %)	n = 12 (24 %)

### Factors influencing transceiver search times and probing time

This study found a significant reduction in search time between the first and the second search trial (means 203.0 s versus 143.2 s; p = 0.002), indicating a significant learning effect even with one single search training. This learning effect was more pronounced with voice compared to no-voice navigation, as total transceiver search time in the second trials was significantly shorter only in the voice navigation group (difference in voice 119.8 s vs. no-voice 164.8 s; p = 0.014). The minimal distance revealed by the transceiver after fine search had a significant influence on the probing time (p = 0.011). A minimal distance of ≤1.1 m resulted in a probing time of mean 44.6 s (SD 5.9 s), whereas minimal distance of >1.1 m resulted in a probing time of mean 116.3 s (SD 32.0 s) p = 0.039). In trials where participants reached a minimal distance of ≤1.1 m also total location time was shorter (44.6 s versus 116.3 s, p = 0.019).

Deviations from the recommended search technique were regularly observed during the search trials, with an incidence varying between 13 % and 55 %. There was no difference in the incidence of major deviations from standard technique between trials with and without voice navigation (Table 2). The influence of each of the nine deviations from the standard search technique on search times is shown in Table 3. The overall rate of deviations from the standard technique per trial was significantly lower in fast compared to slow trials (0.2 deviations per trial in fast trials compared to 0.4 deviations in slow trials, p = 0.028). The error “wrong direction of coarse search” was observed in 55 % of all trials, had a significant impact on the coarse search time (mean 34.1 s vs. 87.3 s with correct direction; p < 0.001), and was corrected earlier in trials with voice compared to no-voice navigation (49.6 s ± 6.9 s versus 93.7 s ± 18.4 s p = 0.011).

**Table 3:** The influence of deviations from the standard search technique on search times. For each deviation this table provides the specific stage of search and the individual influence in the case the deviation was detected versus the correct execution. P-value indicates difference between deviation from standard search and correct search strategy.

Types of Problems and their influence on different stages of search			
Stage of search	**Type of Problem**	**Duration**	**p-value**
Coarse Search	Direction obviously wrong	87.3 ± 10.2 s	p < 0.001
	Correct direction	34.1 ± 4.3 s	

Fine Search	Sudden moves in fine search	76.6 ± 16.6 s	p = 0.013
	Correct fine search	44.9 ± 4.0 s	

Fine Search	No crossing in fine search	69.0 ± 12.8 s	p = 0.014
	Correct fine search	44.2 ± 4.0 s	

Fine Search	Search not on the snow surface	106.6 ± 28.9 s	p = 0.001
	Correct fine search	44.5 ± 3.6 s	

Probing	Probe radius too small	106.4 ± 22.0 s	p < 0.001
	Correct probing	37.5 ± 6.0 s	

Probing	Probing without any scheme	104.6 ± 22.3 s	p = 0.002
	Correct probing	42.7 ± 8.1 s	

Probing	Probing not deep enough	113.6 ± 29.0 s	p = 0.013
	Correct probing	51.1 ± 9.8 s	

## Discussion

This field study demonstrated a wide inter-individual variation in transceiver search times among participants without experience in avalanche rescue. In direct comparison voice navigation did not result in a significant improvement of success rate or total location time. Voice navigation led to a faster correction of a wrong initial direction of coarse search. This study also demonstrated that already a single search training results in a significant reduction in search times and this learning effect was even more pronounced when using voice navigation. All deviations from recommended standard search and probing procedure resulted in a prolongation of search times. Basic training in transceiver search should not only focus on reducing search time but concentrate on the correct performance of the recommended standard search and probing procedure.

The practical advantages of voice navigation are consistent with the fact that in complex and time critical emergency scenarios automated verbal commands are increasingly applied with great success (10). Medical devices, like state-of-the-art automated external defibrillators (AEDs) are fitted with verbal commands, and set an impressive example of enhancing user interactions with a medical device in order to guide unexperienced providers through the various steps of basic life support cardiopulmonary resuscitation (CPR).^18^ The absence of significant improvement of success rate or total location time by using verbal commands in our trial can be explained by current transceiver technology with visual guidance, which can already result in a reasonable performance even in participants without experience.

Participants demonstrated an overall acceptable performance in transceiver search with and without voice navigation despite no practical experience. More than two-thirds were able to locate the victim within 3 min. Our data is in accordance with data from previous field studies demonstrating successful transceiver search within acceptable time limits for unexperienced participants using current, advanced transceiver technology in a simulated avalanche scenario.^19^ On the other hand, the same authors later found out that in real life avalanche rescue search time ranged from 5 to 10 min.^20^, ^21^ Taken together, data obtained during field studies may not correctly reflect performance during a more stressful real life avalanche accident scenario. Furthermore, one should always keep in mind that transceiver search is only one part of avalanche companion rescue. Available data on extrication strategies and shovel techniques after localisation of a totally buried avalanche victim demonstrate that these aspects of companion rescue are markedly more time consuming.^10^, ^22^

Another major finding of the current study was the large inter-individual variability of transceiver search times even though all participants had comparable pre-existing level of training and knowledge in avalanche rescue (i.e. no previous knowledge apart from the pre-test standardised training). Wide inter-individual variations in rescue times were also found in previous studies of simulated companion avalanche rescue.^21^, ^23^ An analysis of faster participants with a total location time < 180 s demonstrated that they made significantly less deviations from the standard search technique. We postulate the existence of large inter-individual differences in the ability to perform transceiver search effectively. Consequently, transceiver search training should not be planned using a fixed time table, but should be individualized, based on individual abilities with the amount of training adopted to learning success.

In the present study a single transceiver search trial reduced search times in the participants by more than one minute, which results in a significantly shorter burial time and a potentially better survival. Comparable to our findings, a previous study found a positive effect of a 15-minute training on the effectiveness of avalanche rescue in otherwise untrained volunteers.^19^, ^21^ Minimal training may not only improve transceiver search times markedly, but also has a significant positive influence on total location time and extrication. This effect was demonstrated after only three consecutive training cycles simulating avalanche extrication (15). Our data suggests that the learning effect of minimal training may even be more pronounced when using voice navigation. In the second trials those participants using voice navigation were significantly faster than those without voice navigation. The results of this study lead to the conclusion that even a device that provides precise and detailed instructions does not replace practical training in which the practice of avalanche rescue is trained in a hands-on manner. Moreover, the difference between voice and non-voice was more pronounced in the second tests, indicating a quantitative interaction between the learning effect and voice, but not a qualitative one. Hence, there appears to be an additional benefit from voice once utilization has been learned and voice navigation helps to increase the learning effect of this training.

We therefore emphasize the importance of regular training in the use of avalanche transceivers and avalanche rescue techniques. The significant improvement in search performance observed in our study after only minimal training may be of particular relevance for mountain guides, who rely on the efficiency of their guests in case of avalanche burial. It is worthwhile to do a short training session with the guests before starting a guided tour to improve the chance of survival in case of avalanche burial.^21^ The importance of improving companion rescue is also related to efficacy of search and rescue intervention. Companion rescue, which is immediate and initiated by those already on the scene, can often make the difference for the efficacy of search and rescue intervention.^11^

Our study showed a close correlation between deviations from standard search procedures and prolonged transceiver search times. Any of the deviations observed from the recommended standard search and probing procedure, resulted in a significant prolongation of search times. Time loss associated with these deviations was also of practical relevance and was in the range of about one minute in many of them. Voice navigation led to a faster correction of a wrong direction of the coarse search. This faster detection and correction may be a key factor to optimize transceiver search in the future. The authors recommend that future basic transceiver search training should not only focus on time measurements, but concentrate on the correct performance of the recommended standard search and probing procedure. Voice navigation and close surveillance of the trainee can help to improve and reinforce the learning process.

The data from the current study underlines the importance of a thorough, precise fine search. Limiting the extent of the area for probing to a minimum with a fine search, reduces the time required for probing by about one minute. Proper and organized probing is also an important determinant of avalanche rescue time. Errors during probing resulted in a mean time loss of about one minute. Our data indicates, that errors and poor performance during probing often resulted in a greater time loss compared to errors and poor performance during transceiver search. The data emphasizes the importance of a coordinated and effective probe training as part of every avalanche transceiver search course.

## Limitations

Despite the authors’ effort to recreate a realistic scenario, this study is still a simulation. We acknowledge the fact that every avalanche rescue scenario is different, and there are many more environmental factors to be considered in real life. The current study was not aimed nor designed to test for training effects, therefore these statements have to be interpreted with caution.

## Conclusion

This study demonstrated a wide inter-individual variation in total transceiver search times and total location time in participants without experience. Voice navigation did not result in a significant reduction of transceiver search times, but led to a faster correction of a wrong initial search direction and improved significantly the learning effect in second trials. Voice navigation could possibly optimize performance in stressful situations for individuals without prior experience. Avalanche rescue training should also concentrate on the avoidance of deviations from the recommended standard procedure during fine search and probing.

## Consent for publication

All participants gave written informed consent to publication of the data.

## CRediT authorship contribution statement

**Bernd Wallner:** Writing – review & editing, Writing – original draft, Visualization, Validation, Supervision, Resources, Project administration, Methodology, Investigation, Funding acquisition, Formal analysis, Data curation, Conceptualization. **Simon Woyke:** Writing – review & editing, Writing – original draft, Validation, Investigation, Formal analysis, Data curation, Conceptualization. **Manuel Winkler:** Writing – review & editing, Writing – original draft, Validation, Methodology, Investigation, Formal analysis, Data curation, Conceptualization. **Fabio Caramazza:** Writing – review & editing, Writing – original draft, Validation, Methodology, Investigation, Formal analysis, Data curation, Conceptualization. **Ivo B. Regli:** Writing – review & editing, Writing – original draft, Validation, Methodology, Investigation, Formal analysis, Data curation, Conceptualization. **Gabriel Putzer:** Writing – review & editing, Writing – original draft, Validation, Supervision, Project administration, Methodology, Investigation, Formal analysis, Data curation, Conceptualization. **Giacomo Strapazzon:** Writing – review & editing, Writing – original draft, Validation, Supervision, Methodology, Investigation, Formal analysis, Data curation, Conceptualization. **Markus Falk:** Writing – review & editing, Writing – original draft, Validation, Software, Methodology, Investigation. **Hermann Brugger:** Writing – review & editing, Writing – original draft, Validation, Supervision, Project administration, Methodology, Investigation, Formal analysis, Data curation, Conceptualization. **Katharina Hüfner:** Writing – review & editing, Writing – original draft, Validation, Supervision, Methodology, Investigation, Formal analysis, Data curation. **Peter Mair:** Writing – review & editing, Writing – original draft, Validation, Supervision, Project administration, Methodology, Investigation, Formal analysis, Data curation, Conceptualization.

## Ethics approval and consent to participate

The ethical committee of the Medical University Innsbruck was consulted, and the participants were informed about the study. The need for ethical approval was waived. All participants provided written informed consent for their participation in the study.

## Funding

BW, FC, SW, MW, PM, GP, and KH are employed by the 10.13039/501100009980Medical University of Innsbruck; IR, GS and HB are employed by 10.13039/100024186Eurac Research Bolzano; and MF is self-employed. None of these sponsors had any role in the study design, the collection, analysis or interpretation of the data, the writing of the manuscript, or the decision to submit the manuscript. The Ortovox company provided non-financial support. There was no other funding supporting this manuscript.

## Declaration of competing interest

The authors declare that they have no known competing financial interests or personal relationships that could have appeared to influence the work reported in this paper.

## References

[1] Falk M., Brugger H., Adler-Kastner L. (1994). Avalanche survival chances. Nature.

[2] Brugger H., Etter H.J., Boyd J., Falk M. (2009). Causes of death from avalanche. Wilderness Environ Med.

[3] Haegeli P., Falk M., Procter E. (2014). The effectiveness of avalanche airbags. Resuscitation.

[4] Procter E., Strapazzon G., Dal Cappello T. (2016). Burial duration, depth and air pocket explain avalanche survival patterns in Austria and Switzerland. Resuscitation.

[5] Strapazzon G., Taboni A., Dietrichs E.S., Luks A.M., Brugger H. (2024). Avalanche burial pathophysiology - a unique combination of hypoxia, hypercapnia and hypothermia. J Physiol.

[6] Haegeli P., Falk M., Brugger H., Etter H.J., Boyd J. (2011). Comparison of avalanche survival patterns in Canada and Switzerland. CMAJ.

[7] Brugger H., Durrer B., Adler-Kastner L., Falk M., Tschirky F. (2001). Field management of avalanche victims. Resuscitation.

[8] Rauch S., Brugger H., Falk M. (2024). Avalanche survival rates in Switzerland, 1981-2020. JAMA Netw Open.

[9] Brugger H., Sumann G., Meister R. (2003). Hypoxia and hypercapnia during respiration into an artificial air pocket in snow: implications for avalanche survival. Resuscitation.

[10] Slotta-bachmayr L. (2005). How burial time of avalanche victims is influenced by rescue method: an analysis of search reports from the Alps. Nat Hazards J Int Soc Prevent Mitig Nat Hazards.

[11] Mair P., Frimmel C., Vergeiner G. (2013). Emergency medical helicopter operations for avalanche accidents. Resuscitation.

[12] Brugger H., Etter H.J., Zweifel B. (2007). The impact of avalanche rescue devices on survival. Resuscitation.

[13] Ng P., Smith W.R., Wheeler A., McIntosh S.E. (2015). Advanced avalanche safety equipment of backcountry users: current trends and perceptions. Wilderness Environ Med.

[14] Procter E., Strapazzon G., Dal Cappello T., Castlunger L., Staffler H.P., Brugger H. (2014). Adherence of backcountry winter recreationists to avalanche prevention and safety practices in northern Italy. Scand J Med Sci Sports.

[15] Wallner B., Caramazza F., Woyke S. (2025). The effect of automated verbal commands during avalanche transceiver search on acute Mental stress and arousal-a mixed-methods crossover field study. Brain Behav.

[16] Ortovox Sportartikel GmbH. Ortovox Diract Voice avalanche transceiver. Available from https://www.ispo.com/en/promotion-ortovox/ortovox-avalanche-transceiver-voice-navigation-diract-voice. Accessed 07 November 2024.

[17] Austrian Alpine Club, Österreichischer Alpenverein. Available from https://www.youtube.com/watch?v=-ZvRMTJdj4c. Accessed 07 November 2024.

[18] Beckers S., Fries M., Bickenbach J., Derwall M., Kuhlen R., Rossaint R. (2005). Minimal instructions improve the performance of laypersons in the use of semiautomatic and automatic external defibrillators. Crit Care.

[19] Genswein M.E., Ragnhild E. (2008). Proceedings of the international snow science workshop, Whistler, BC, Canada.

[20] Genswein M.E., Ragnhild E. (2008). Proceedings of the international snow science workshop, Whistler, BC, Canada.

[21] Genswein M. (2009). Proceedings of the international snow science workshop 2009, Davos, Switzerland.

[22] Wallner B., Moroder L., Brandt A. (2019). Extrication times during avalanche companion rescue: a randomized single-blinded manikin study. High Alt Med Biol.

[23] Edgerly B., Atkins D., Recco A.B. (2006,). Proceedings of the international snow science workshop 2006.

