# Supplementary material for: Avalanche transceiver search times during avalanche companion rescue – A prospective randomized single-blinded cross-over simulation study
Source: Resusc Plus. 2025 Aug 19;26:101065. doi: 10.1016/j.resplu.2025.101065 (PMC12415072; doi:10.1016/j.resplu.2025.101065)
Supplement: Supplementary Data 1 [file mmc1.docx]

Supplemental material: Key Elements to Report for Simulation-Based Research

| **Elements** | **Subelements** | **Descriptor** |
| --- | --- | --- |
| Participant orientation | Orientation to the simulator | In order to make the scenario as realistic as possible, we deliberately decided not to provide any orientation or familiarization with the simulation situation. |
|  | Orientation to the environment | The study participants were given a link to an instructional video and were free to move around the ski room before the study was conducted. However, a conscious decision was made not to allow the study participants to see the simulation situation or prepare for it. |
| Simulator type | Simulator make and model | The simulation scenario was a realistic reenactment of an avalanche field in which a ski touring companion was buried. A great deal of effort was put into recreating as realistic a burial situation as possible. |
|  | Simulator functionality | A test setup was constructed in advance of the study to simulate the conditions. Two transmitter plates were buried exactly one meter below the surface (Figure 1). The participants started from the same starting position and were equipped with either a transceiver with or without voice navigation. |
| Simulation environment | Location | The location was situated at 2450 m a.s.l. in the Kühtai skiing area, Tyrol, Austria. An artificial avalanche field was prepared on a level surface with two transmitters buried under the snow-surface (RTX457 Mobile Avalanche Transceiver Training System; developed by Girsberger Mountain Rescue Technology). |
|  | Equipment | Avalanche transmitters: RTX457 Mobile Avalanche Transceiver Training System; developed by Girsberger Mountain Rescue Technology  Avalanche transceivers: Ortovox, diract voice avalanche transceiver, Ortovox Sportartikel GmbH, Rotwandweg 3a, D-82024 Taufkirchen, Germany |
|  | External stimuli | We designed the simulation to be as realistic as possible including realistic external stimuli. The objective environmental parameters during the course of the experiments were the meteorological conditions (mostly sunny, temperature during the experiment 2°C – 7° C; hardly any wind), high altitude and physical exertion. The condition of the snow (the snow density had a median of 320 kg/m³) can be compared to the avalanche debris. |
| Simulation event/scenario | Event description | One week prior to the trials, a video with structured instructions on how to perform an avalanche search and how to adequately use a standard avalanche transceiver (not the ones used in the experiment) and probe was sent to each participant. Immediately prior to each trial, the participants were confronted with the hypothetical scenario of being part of a skiing team, and one of their teammates was buried by an avalanche to simulate a realistic scenario. All participating researchers were instructed to only interact with participants in a very professional and distant manner and to refrain from any encouragement or small talk with the participants in order not to influence stress levels through human interaction. |
|  | Learning objectives | There were no learning objectives identified. |
|  | Group vs. individual practice | The simulation was conducted as individuals. |
|  | Use of adjuncts | No adjuncts (e.g., moulage, media, props) were used. |
|  | Facilitator/operator characteristics | The study team consisted of specialists in anesthesiology, psychiatry, and emergency medicine. The group of study collaborators has extensive expertise in the field of medical education and simulation training. |
|  | Pilot testing | Exactly three weeks before the study was conducted, the study team carried out a pilot test with test subjects. A total of four test runs were carried out and minor adjustments were made to the study procedure. |
|  | Actors/confederates/standardized/ simulated patients | Not applicable |
| Instructional design (for educational interventions) or exposure (for simulation as investigative methodology) | Duration | A video with structured instructions on how to perform an avalanche search and how to adequately use a standard avalanche transceiver (not the ones used in the experiment) and probe was sent to each participant. Immediately prior to each trial, the participants were confronted with the hypothetical scenario of being part of a skiing team, and one of their teammates was buried by an avalanche to simulate a realistic scenario. |
|  | Timing | One week prior to the trials |
|  | Frequency/repetitions | Each participant performed two trials |
|  | Clinical variation | The scenario was identical each time, the only variation included the transmitter location and the avalanche transceiver (one with and one without voice navigation). |
|  | Standards/assessment | The only standard that was defined was the requirement that all participants and no prior training in avalanche rescue and no experience with an avalanche transceiver. |
|  | Adaptability of intervention | The scenario was not responsive to individual learner needs in any way and was conducted in completely the same way each time. |
|  | Range of difficulty | There was absolutely no variation in difficulty or complexity of the task. |
|  | Nonsimulation interventions  and adjuncts | Each trial was followed by a standardized psychological questionnaire and A personal, semistructured interview was performed with each participant after the completion of both trials by authors F.C. and K.H [16]. The participants were asked about the detailed aspects of the effects that voice navigation had on the perceived levels of mental stress, arousal and performance [16]. |
|  | Integration | The intervention was not integrated into any curriculum. |
| Feedback and/or debriefing | Source | Each trial was followed by a short questionnaire. After completion of the entire study (both test trials), an individualized and standardized debriefing was conducted, followed by a semi-structured interview. This debriefing was conducted by a study staff member, who answered all questions from the study participants and provided tips for improving performance. However, this took place only after the study was completed and outside of the study, and served only to help the study participants improve personally. |
|  | Duration | The duration of the debriefing was adapted to the needs of each participant and lasted from 5 to 20 minutes. |
|  | Facilitator presence | For each debriefing there was only one (F.C. or K.H) facilitator present. |
|  | Facilitator characteristics | F.C. is an experienced anesthesiologist, intensive care physician, and emergency physician who has already collaborated on several similar studies in the Alps.  K.H. is a specialist in neurology and psychiatry with extensive experience in conducting similar studies and psychological testing. |
|  | Content/  Structure/method | After the semi-structured interview, an individualized debriefing took place in which the personal performance of each participant was discussed. Special attention was paid to the search strategy and probing. Questions were answered and each participant was given tips on how to improve their search strategy. |
|  | Timing | The interview and the debriefing was performed immediately after the last trial. |
|  | Video | There was no video used in any form. |
|  | Scripting | There was no scripting used in any form. |
