# Supplementary material for: Avalanche transceiver search times during avalanche companion rescue – A prospective randomized single-blinded cross-over simulation study
Source: Resusc Plus. 2025 Aug 19;26:101065. doi: 10.1016/j.resplu.2025.101065 (PMC12415072; doi:10.1016/j.resplu.2025.101065)
Supplement: Supplementary Data 2 [file mmc2.pdf]

Categorization of Simulation Research Checklist

Use of Simulation		Example
	**1.** □ Simulation as Training mechanism	A prospective study of the effect of a novel simulation-based medical education intervention on resident performance in medical crises
	**2.** □ Simulation as Investigative Methodology or Environment of Research	A study of communication pattern differences between surgeons and nursing staff of differing seniority using a simulated environment to recreate relevant clinical situations.
**Goal of Research**		
	**1.** □ Assessment	A study assessing healthcare provider competence in sedation with the use of simulation-based methodologies.
	**2.** □ Learning Outcomes	A study examining the knowledge and skills acquired by learners during a simulation of pediatric resuscitation
	**3.** □ Translational Outcomes	A study examining the effect of a simulation-based intervention designed to address rapid dysrhythmia recognition on dysrhythmia-based cardiac arrest survival.
	**4.** □ Instructional Design	A study examining whether a difference in learning occurs depending on whether a simulation session uses traditional post-case debriefing or a “stop and go” debriefing (debriefing conducted at regular intervals throughout the case) format.
	**5.** □ Systems probing	A study of hospital code team preparedness using in-situ unannounced code simulations as a testing environment.
	**6.** □ Technology Testing	As study evaluating the use of a novel chest-tube placement trainer and its effect on provider technical skills

This checklist lists the major categories of simulation research and provides clarifying examples. Novice researchers are encouraged to use this as a checklist when considering the overall category of simulation research into which their specific interests fall.

For additional relevant literature, please see Calhoun et al. Empowering the Inexperienced Researcher: A Summary Report and Expert Recommendations; available on the Society for Simulation in Healthcare Research Portal (www.ssih.org).
