# Supplementary material for: Avalanche transceiver search times during avalanche companion rescue – A prospective randomized single-blinded cross-over simulation study
Source: Resusc Plus. 2025 Aug 19;26:101065. doi: 10.1016/j.resplu.2025.101065 (PMC12415072; doi:10.1016/j.resplu.2025.101065)
Supplement: Supplementary Data 3 [file mmc3.pdf]

cs-author@wiley.com <cs-author@wiley.com> 12.8.2025 10:59

[EXTERN] Permission from the publisher Your case 26979747 [ref:!00Dd00eeku.!500WQ0u16uO:ref]

An bernd.wallner@i-med.ac.at <bernd.wallner@i-med.ac.at> Kopie onbehalfof@manuscriptcentral.com <onbehalfof@manuscriptcentral.com> • agrethe@ucsd.edu <agrethe@ucsd.edu> • brain@wiley.com <brain@wiley.com>

Dear Bernd Wallner,

Journal: Brain and Behavior
“The effect of automated verbal commands during avalanche transceiver search on acute mental stress and arousal – a mixed methods crossover field study”
Article ID: BRB370684

Thank you for your recent communication requesting permission to use material from one of our publications.

We are pleased to inform you that since the article is published under the CC-BY license, you are allowed to use your article contents including figures.

For more information on the CC-BY license please visit https://creativecommons.org/licenses/by/4.0/.

Please do not hesitate to contact us again if you require further assistance.

Kind regards,

Cynthia Redoma
Wiley Author Support

WILEY

If you require further assistance with this matter or would like answers to frequently asked questions, please visit Wiley Author Support, 24 hours a day, 7 days a week.

--------------- Original Message ---------------
From: Bernd Wallner [bernd.wallner@i-med.ac.at]
Sent: 8/12/2025, 2:56 PM
To: cs-author@wiley.com; brain@wiley.com; agrethe@ucsd.edu; onbehalfof@manuscriptcentral.com
Subject: Permission from the publisher [Case Number : 26979747] {500WQ00000u16uOYAQ. 003WQ00000iJFZtYAO}

This is an external email.

Dear Ladies and Gentlemen,

we have recently published our article in your journal Brain and Behavior.

Article ID: BRB370684
Article DOI: 10.1002/brb3.70684
Internal Article ID: 100282846
Article: The effect of automated verbal commands during avalanche transceiver search on acute mental stress and arousal – a mixed methods crossover field study
Journal: Brain and Behavior

We have further submitted the second part of this article, with completely different data and completely new results to the Journal Resuscitation PLUS.

However we would like to use Figure 1 from the already published article and would like to obtain the "Permission from the publisher" and the right that Figure 1 may also be published in this second part of the study in the Journal Resuscitation PLUS.

We are sure and certain that the double use of this Figure does not in any way interfere with the already published data or article.
Figure 1 merely explains the setup of the study.

Thank you very much for granting us this permission.

Kind regards,
Bernd Wallner

Dr. med. univ. Bernd Wallner PhD EDAIC

Medical University Innsbruck
Department of Anaesthesiology and Intensive Care Medicine

Anichstraße 35, 6020 Innsbruck
+43/512/504/80858
bernd.wallner@i-med.ac.at
www.i-med.ac.at

Diese Nachricht und allfällige angehängte Dokumente sind vertraulich und nur für den/die Adressaten bestimmt. Sollten Sie nicht der beabsichtigte Adressat sein, ist jede Offenlegung, Weiterleitung oder sonstige Verwendung dieser Information nicht gestattet. In diesem Fall bitten wir, den Absender zu verständigen und die Information zu vernichten. Für Übermittlungsfehler oder sonstige Irrtümer bei Übermittlung besteht keine Haftung.

This message and any attached files are confidential and intended solely for the addressee(s). Any publication, transmission or other use of the information by a person or entity other than the intended addressee is prohibited. If you receive this in error please contact the sender and delete the material. The sender does not accept liability for any errors or omissions as a result of the transmission.

ref:!00Dd00eeku.!500WQ0u16uO:ref
